# Supplementary material for: Target-enriched enzymatic methyl sequencing: Flexible, scalable and inexpensive hybridization capture for quantifying DNA methylation
Source: PLoS One. 2023 Mar 9;18(3):e0282672. doi: 10.1371/journal.pone.0282672 (PMC9997987; doi:10.1371/journal.pone.0282672)
Supplement: S1 Fig — Integrative Genomics Viewer (IGV) logarithm-scale coverage and read alignment plots (for deduplicated data) of superb starling sample BB-17168 multi-exonic targets for (a) FK506 binding protein 5 (FKBP5), (b) the glucocorticoid receptor (NR3C1), and (c) the androgen receptor (AR). FKBP5 includes the entire promoter and gene region, whereas NR3C1 and AR are focused on the end of each gene region to enable IGV’s display of read alignments. Alignments are colored in bisulfite mode by CG, with red indicating methylated sites (non-converted cytosines) and blue indicating unmethylated sites (converted cytosines). Target tracks for the superb starling genome assembly (updated chromosomal-level genome assembly: CU_Lasu_v2; GenBank: GCA_015883425.2) include GenBank sequence ID, locus tag, and protein IDs (with exonic spans also in blue). (DOCX) [file pone.0282672.s001.docx]

**S1 Fig.** **Representative examples of target-enriched enzymatic methyl sequencing (TEEM-Seq) versus whole-genome enzymatic methyl sequencing (WGEM-Seq) coverage**. Integrative Genomics Viewer (IGV) logarithm-scale coverage and read alignment plots (for deduplicated data) of superb starling sample BB-17168 multi-exonic targets for (**a**) FK506 binding protein 5 (*FKBP5*), (**b**) the glucocorticoid receptor (*NR3C1*), and (**c**) the androgen receptor (*AR*). *FKBP5* includes the entire promoter and gene region, whereas *NR3C1* and *AR* are focused on the end of each gene region to enable IGV's display of read alignments. Alignments are colored in bisulfite mode by CG, with red indicating methylated sites (non-converted cytosines) and blue indicating unmethylated sites (converted cytosines). Target tracks for the superb starling genome assembly (updated chromosomal-level genome assembly: CU_Lasu_v2; GenBank: GCA_015883425.2) include GenBank sequence ID, locus tag, and protein IDs (with exonic spans also in blue).
