# Supplementary material for: Target-enriched enzymatic methyl sequencing: Flexible, scalable and inexpensive hybridization capture for quantifying DNA methylation
Source: PLoS One. 2023 Mar 9;18(3):e0282672. doi: 10.1371/journal.pone.0282672 (PMC9997987; doi:10.1371/journal.pone.0282672)
Supplement: S2 Fig — CpG sites shared between TEEM-Seq libraries at 5x coverage or above were used for paired comparisons within samples with the getCorrelation function in methylKit: (a) BB-17411 has 5,073 CpG sites shared with its duplicate library; (b) BB-17455 has 5,034 CpG sites shared with its duplicate library; (c) B-40881 has CpG 5,410 sites shared with its duplicate library; and (d) BB-17532 has 4,867 CpG sites shared with its first duplicate library, (e) 4,309 CpG sites shared with its second duplicate library, and (e) 4,248 CpG sites shared between the first and second duplicate libraries. Blue regions of the scatterplots are uncorrelated, yellow regions are highly correlated, and green regions are variably correlated. The green lines represents lowess polynomial regression fits, whereas the red lines represent linear regression fits. (DOCX) [file pone.0282672.s002.docx]

**S2 Fig. Putative promoter target region CpG site-level percent DNA methylation distribution histograms for each sample library and scatterplots with Pearson correlation coefficients between target-enriched enzymatic methyl sequencing (TEEM-Seq) duplicate and triplicate libraries for superb starling individuals BB-17411, BB-17455, B-40881, and BB-17532.** CpG sites shared between TEEM-Seq libraries at 5x coverage or above were used for paired comparisons within samples with the *getCorrelation* function in methylKit: (a) BB-17411 has 5,073 CpG sites shared with its duplicate library; (b) BB-17455 has 5,034 CpG sites shared with its duplicate library; (c) B-40881 has CpG 5,410 sites shared with its duplicate library; and (d) BB-17532 has 4,867 CpG sites shared with its first duplicate library, (e) 4,309 CpG sites shared with its second duplicate library, and (e) 4,248 CpG sites shared between the first and second duplicate libraries. Blue regions of the scatterplots are uncorrelated, yellow regions are highly correlated, and green regions are variably correlated. The green lines represents lowess polynomial regression fits, whereas the red lines represent linear regression fits.
