# Supplementary material for: Target-enriched enzymatic methyl sequencing: Flexible, scalable and inexpensive hybridization capture for quantifying DNA methylation
Source: PLoS One. 2023 Mar 9;18(3):e0282672. doi: 10.1371/journal.pone.0282672 (PMC9997987; doi:10.1371/journal.pone.0282672)
Supplement: S3 Fig — Libraries for three individual superb starlings (BB-17168 [green triangles], BB-17501 [blue squares], and BB-14232 [red circles]) were analyzed with and without deduplication. Mean DNA methylation by putative promoter target region for deduplication and non-deduplication of samples were similar (the mean absolute difference across genes was 0.77 for BB-17168, 0.78 for BB-17501, and 0.89 for BB-14232). To parallel Fig 5a, CpG site-level Pearson correlations were also analyzed using the same sites for BB-17168 (R = 0.99, N = 2733, P < 0.0001), BB-17501 (R = 0.99, N = 2770, P < 0.0001), and BB-14232 (R = 0.99, N = 1588, P < 0.0001). (DOCX) [file pone.0282672.s003.docx]

**
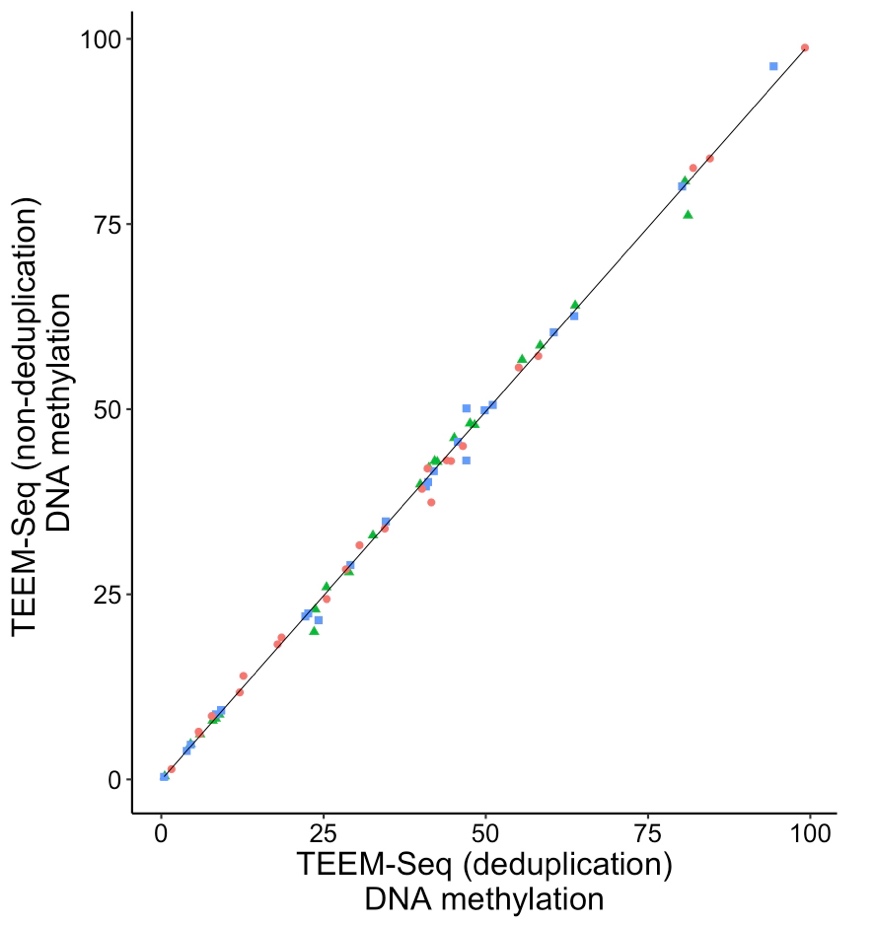
**

**S3 Fig. Comparison of target-enriched enzymatic methyl sequencing (TEEM-Seq) data with and without deduplication.** Libraries for three individual superb starlings (BB-17168 [green triangles], BB-17501 [blue squares], and BB-14232 [red circles]) were analyzed with and without deduplication. Mean DNA methylation by putative promoter target region for deduplication and non-deduplication of samples were similar (the mean absolute difference across genes was 0.77 for BB-17168, 0.78 for BB-17501, and 0.89 for BB-14232). To parallel Fig 5a, CpG site-level Pearson correlations were also analyzed using the same sites for BB-17168 (R = 0.99, N = 2733, P < 0.0001), BB-17501 (R = 0.99, N = 2770, P < 0.0001), and BB-14232 (R = 0.99, N = 1588, P < 0.0001).
