# Supplementary material for: Target-enriched enzymatic methyl sequencing: Flexible, scalable and inexpensive hybridization capture for quantifying DNA methylation
Source: PLoS One. 2023 Mar 9;18(3):e0282672. doi: 10.1371/journal.pone.0282672 (PMC9997987; doi:10.1371/journal.pone.0282672)
Supplement: S4 Fig — CpG sites shared between TEEM-Seq and WGEM-Seq libraries at 5x coverage or above were used for paired comparisons within samples with the getCorrelation function in methylKit for (a) BB-17168 (2,732 CpG sites shared), (b) BB-17501 (2,769 CpG sites shared), and (c) BB-14232 (1,587 CpG sites shared). Blue regions of the scatterplots are uncorrelated, yellow regions are highly correlated, and green regions are variably correlated. The green lines represents lowess polynomial regression fits, whereas the red lines represent linear regression fits. (DOCX) [file pone.0282672.s004.docx]

**S4 Fig. Putative promoter target region CpG site-level percent DNA methylation distribution histograms for each sample library and scatterplots with Pearson correlation coefficients between target-enriched enzymatic methyl sequencing (TEEM-Seq) and whole-genome enzymatic methyl sequencing (WGEM-Seq) libraries from the same individual superb starlings.** CpG sites shared between TEEM-Seq and WGEM-Seq libraries at 5x coverage or above were used for paired comparisons within samples with the *getCorrelation* function in methylKit for (a) BB-17168 (2,732 CpG sites shared), (b) BB-17501 (2,769 CpG sites shared), and (c) BB-14232 (1,587 CpG sites shared). Blue regions of the scatterplots are uncorrelated, yellow regions are highly correlated, and green regions are variably correlated. The green lines represents lowess polynomial regression fits, whereas the red lines represent linear regression fits.
