# Supplementary material for: Target-enriched enzymatic methyl sequencing: Flexible, scalable and inexpensive hybridization capture for quantifying DNA methylation
Source: PLoS One. 2023 Mar 9;18(3):e0282672. doi: 10.1371/journal.pone.0282672 (PMC9997987; doi:10.1371/journal.pone.0282672)
Supplement: S5 Fig — CpG sites shared at 5x coverage or above were used for paired comparisons with the getCorrelation function in methylKit. (a) Comparisons of the entire genome at a 5x threshold for CpG site coverage include 271,828 sites shared across all libraries, whereas (b) comparisons at a 7x threshold include 37,611 CpG sites shared across all libraries. Blue regions of the scatterplots are uncorrelated, yellow regions are highly correlated, and green regions are variably correlated. The green lines represents lowess polynomial regression fits, whereas the red lines represent linear regression fits. (DOCX) [file pone.0282672.s005.docx]

**S5 Fig. Comparisons of CpG site-level percent DNA methylation distribution histograms for each sample library and scatterplots with Pearson correlation coefficients of whole-genome enzymatic methyl sequencing (WGEM-Seq) and reduced-representation bisulfite sequencing (RRBS) libraries from the same three individual superb starlings: BB-17168, BB-17501, and BB-14232.** CpG sites shared at 5x coverage or above were used for paired comparisons with the *getCorrelation* function in methylKit. (a) Comparisons of the entire genome at a 5x threshold for CpG site coverage include 271,828 sites shared across all libraries, whereas (b) comparisons at a 7x threshold include 37,611 CpG sites shared across all libraries. Blue regions of the scatterplots are uncorrelated, yellow regions are highly correlated, and green regions are variably correlated. The green lines represents lowess polynomial regression fits, whereas the red lines represent linear regression fits.
