# Supplementary material for: Target-enriched enzymatic methyl sequencing: Flexible, scalable and inexpensive hybridization capture for quantifying DNA methylation
Source: PLoS One. 2023 Mar 9;18(3):e0282672. doi: 10.1371/journal.pone.0282672 (PMC9997987; doi:10.1371/journal.pone.0282672)
Supplement: S1 Table — (DOCX) [file pone.0282672.s007.docx]

**S1 Table. Location of putative promoter bait targets in the superb starling reference genome.**

| **Target region** | **Putative promoter region (bp)** | **Genome position** | | **Putative promoter bait target** | |
| --- | --- | --- | --- | --- | --- |
|  |  | **CU_Lasu_v2 sequence** | **Chromosome** | **Start sequence location**  **(1-based)** | **End sequence location** |
| *AR* | 4000 | CM040307.1 | 4A | 14532331 | 14536330 |
| *AVPR1A* | 4000 | CM040303.1 | 1A | 45531591 | 45535590 |
| *AVPR1B* | 4000 | CM040330.1 | 26 | 5619147 | 5623146 |
| *CRH* | 4000 | CM040304.1 | 2 | 116333274 | 116337273 |
| *DNMT1* | 4000 | JADDUC020000037.1 | N/A | 142444 | 146443 |
| *DNMT3A* | 4000 | CM040305.1 | 3 | 111778701 | 111782700 |
| *DNMT3B* | 4000 | CM040324.1 | 20 | 6045685 | 6049684 |
| *EGR1* | 4000 | CM040318.1 | 13 | 19257051 | 19261050 |
| *ESR1* | 4000 | CM040305.1 | 3 | 54837600 | 54841599 |
| *FKBP5* | 4000 | CM040330.1 | 26 | 2992197 | 2996196 |
| *GNIH* | 4000 | CM040304.1 | 2 | 32620135 | 32624134 |
| *GNRH1* | 5726* | CM040326.1 | 22 | 2727612 | 2733338 |
| *GNRHR2 r1*^#^ | 4000 | CM040315.1 | 10 | 17836010 | 17840009 |
| *GNRHR2 r2*^#^ | 4000 | CM040315.1 | 10 | 20554109 | 20558108 |
| *MC2R* | 3856 | CM040304.1 | 2 | 96963447 | 96967302 |
| *MC4R* | 3948* | CM040304.1 | 2 | 70409831 | 70413779 |
| *NR3C1* | 4000 | CM040318.1 | 13 | 18404677 | 18408676 |
| *NR3C2* | 4000 | CM040306.1 | 4 | 59434518 | 59438517 |
| *OXTR* | 4000 | CM040317.1 | 12 | 20204345 | 20208344 |
| *POMC* | 258** | JADDUC020000174.1 | N/A | 1 | 258 |
| *SERPINA1* | 4000 | CM040308.1 | 5A | 15667895 | 15671894 |
| *VT* | 4000 | CM040306.1 | 4 | 3393601 | 3397600 |
| *VTG1* | 697** | CM040313.1 | 8 | 18775171 | 18775867 |

^*^ Since there is no existing annotation in the superb starling reference genome for this gene, we used the zebra finch sequence alignment to target 2 kb upstream in the putative promoter region and 2 kb in the gene body.

^**^ Limited sequence upstream of gene.

^#^ Two separate gene regions (indicated as r1 and r2) on chromosome 10 with similarity to *GNRHR2*.
