# Supplementary material for: Target-enriched enzymatic methyl sequencing: Flexible, scalable and inexpensive hybridization capture for quantifying DNA methylation
Source: PLoS One. 2023 Mar 9;18(3):e0282672. doi: 10.1371/journal.pone.0282672 (PMC9997987; doi:10.1371/journal.pone.0282672)
Supplement: S2 Table — (DOCX) [file pone.0282672.s008.docx]

**S2 Table. Location of exon bait targets in the superb starling reference genome.**

| **Target region** | **Genome position**  ***CU_Lasu_v2 sequence*** | **Exon start sequence location**  **(1-based)** | **Exon end sequence location** |
| --- | --- | --- | --- |
| *AR* | CM040307.1 | 14536331 | 14536608 |
| *AR* | CM040307.1 | 14553510 | 14553661 |
| *AR* | CM040307.1 | 14561214 | 14561330 |
| *AR* | CM040307.1 | 14566681 | 14566968 |
| *AR* | CM040307.1 | 14569050 | 14569194 |
| *AR* | CM040307.1 | 14569959 | 14570089 |
| *AR* | CM040307.1 | 14570290 | 14570447 |
| *AR* | CM040307.1 | 14570702 | 14570827 |
| *AR* | CM040307.1 | 14571069 | 14571192 |
| *AR* | CM040307.1 | 14571221 | 14571654 |
| *AR* | CM040307.1 | 14571708 | 14572081 |
| *AR* | CM040307.1 | 14572630 | 14572735 |
| *AVPR1A* | CM040303.1 | 45528656 | 45528936 |
| *AVPR1A* | CM040303.1 | 45530634 | 45531541 |
| *AVPR1A* | CM040303.1 | 45531556 | 45531590 |
| *AVPR1B* | CM040330.1 | 5617298 | 5617587 |
| *AVPR1B* | CM040330.1 | 5618209 | 5619146 |
| *CRH* | CM040304.1 | 116332740 | 116333273 |
| *EGR1* | CM040318.1 | 19261051 | 19261285 |
| *EGR1* | CM040318.1 | 19261584 | 19262881 |
| *FKBP5* | CM040330.1 | 2996197 | 2996314 |
| *FKBP5* | CM040330.1 | 2997227 | 2997320 |
| *FKBP5* | CM040330.1 | 2997561 | 2997662 |
| *FKBP5* | CM040330.1 | 2998610 | 2998702 |
| *FKBP5* | CM040330.1 | 3001834 | 3001990 |
| *FKBP5* | CM040330.1 | 3002418 | 3002508 |
| *FKBP5* | CM040330.1 | 3003256 | 3003339 |
| *FKBP5* | CM040330.1 | 3003787 | 3003920 |
| *FKBP5* | CM040330.1 | 3005358 | 3005553 |
| *GNRHR2 r1* | CM040315.1 | 17840010 | 17840015 |
| *GNRHR2 r1* | CM040315.1 | 17840516 | 17841025 |
| *GNRHR2 r1* | CM040315.1 | 17841058 | 17841302 |
| *GNRHR2 r1* | CM040315.1 | 17841288 | 17841489 |
| *GNRHR2 r1* | CM040315.1 | 17841772 | 17842178 |
| *GNRHR2 r1* | CM040315.1 | 17842084 | 17842174 |
| *GNRHR2 r2* | CM040315.1 | 20552342 | 20553124 |
| *GNRHR2 r2* | CM040315.1 | 20553536 | 20554108 |
| *NR3C1* | CM040318.1 | 18337403 | 18340650 |
| *NR3C1* | CM040318.1 | 18340700 | 18340793 |
| *NR3C1* | CM040318.1 | 18341168 | 18341240 |
| *NR3C1* | CM040318.1 | 18341319 | 18341645 |
| *NR3C1* | CM040318.1 | 18351707 | 18351837 |
| *NR3C1* | CM040318.1 | 18355842 | 18356086 |
| *NR3C1* | CM040318.1 | 18359382 | 18359539 |
| *NR3C1* | CM040318.1 | 18365651 | 18365767 |
| *NR3C1* | CM040318.1 | 18369957 | 18370120 |
| *NR3C1* | CM040318.1 | 18403494 | 18404676 |
| *NR3C2* | CM040306.1 | 59422547 | 59422562 |
| *NR3C2* | CM040306.1 | 59430594 | 59432361 |
| *NR3C2* | CM040306.1 | 59433268 | 59433334 |
| *NR3C2* | CM040306.1 | 59434509 | 59434517 |
| *OXTR* | CM040317.1 | 20203310 | 20204344 |
| *POMC* | JADDUC020000174.1 | 259 | 552 |
