# Supplementary material for: Target-enriched enzymatic methyl sequencing: Flexible, scalable and inexpensive hybridization capture for quantifying DNA methylation
Source: PLoS One. 2023 Mar 9;18(3):e0282672. doi: 10.1371/journal.pone.0282672 (PMC9997987; doi:10.1371/journal.pone.0282672)
Supplement: S3 Table — TEEM-Seq alignments were performed on target region putative promoters and exons to generate mean coverage plots (Fig 2). Since some exon spans were too short for proper alignments, reads were also aligned separately with neighboring intron sequence for proper alignments and then checked for exon depth (see Fig 2d–2f). There were 106,178 total bases in the target sequences. (DOCX) [file pone.0282672.s009.docx]

**S3 Table. Summary metrics for target-enriched enzymatic methyl sequencing (TEEM-Seq).** TEEM-Seq alignments were performed on target region putative promoters and exons to generate mean coverage plots (Fig 2). Since some exon spans were too short for proper alignments, reads were also aligned separately with neighboring intron sequence for proper alignments and then checked for exon depth (see Fig 2d-f). There were 106,178 total bases in the target sequences.

| **Individual** | **Raw reads** | **Target region mapping efficiency (%)** | **Target region unique paired-end alignments** | **Target region sequences after deduplication** | **Target region mapped reads after deduplication** | **Mean read depth across putative promoter target regions** |
| --- | --- | --- | --- | --- | --- | --- |
| BB-17532 | 14,423,547 | 13.7 | 1,965,052 | 124,628 | 249,256 | 318.54x |
| BB-17501 | 10,238,150 | 14.3 | 1,459,928 | 36,825 | 73,650 | 94.44x |
| BB-17455 | 12,105,745 | 16.4 | 1,981,679 | 98,146 | 196,292 | 254.84x |
| BB-17411 | 12,538,149 | 15.5 | 1,934,046 | 68,158 | 136,316 | 178.43x |
| BB-17168 | 5,905,164 | 15.6 | 917,093 | 25,943 | 51,886 | 65.73x |
| BB-14232 | 2,777,787 | 15.9 | 440,740 | 20,203 | 40,406 | 51.6x |
| B-40881 | 37,427,806 | 16.7 | 6,209,452 | 309,228 | 618,456 | 772.53x |
