# Supplementary material for: Target-enriched enzymatic methyl sequencing: Flexible, scalable and inexpensive hybridization capture for quantifying DNA methylation
Source: PLoS One. 2023 Mar 9;18(3):e0282672. doi: 10.1371/journal.pone.0282672 (PMC9997987; doi:10.1371/journal.pone.0282672)
Supplement: S4 Table — WGEM-Seq alignments were performed on target region putative promoters and exons to generate mean coverage plots (Fig 2). Since some exon spans were too short for proper alignments, reads were also aligned separately with neighboring intron sequence for proper alignments and then checked for exon depth (see Fig 2d–2f). There were 106,178 total bases in the target sequences. (DOCX) [file pone.0282672.s010.docx]

**S4 Table. Summary metrics for whole-genome enzymatic methyl sequencing (WGEM-Seq).** WGEM-Seq alignments were performed on target region putative promoters and exons to generate mean coverage plots (Fig 2). Since some exon spans were too short for proper alignments, reads were also aligned separately with neighboring intron sequence for proper alignments and then checked for exon depth (see Fig 2d-f). There were 106,178 total bases in the target sequences.

| **Individual** | **Raw reads** | **Full genome mapping efficiency (%)** | **Full genome unique paired-end alignments** | **Full genome sequences after deduplication** | **Mapped reads after deduplication** | **Mean read depth across genome** | **Mean read depth across putative promoter target regions** |
| --- | --- | --- | --- | --- | --- | --- | --- |
| BB-17501 | 122,075,233 | 73.8 | 89,559,902 | 75,747,783 | 151,495,566 | 23.07x | 16.04x |
| BB-17168 | 134,336,464 | 73.2 | 97,542,032 | 80,729,566 | 161,459,132 | 26.03x | 17.71x |
| BB-14232 | 92,367,134 | 75.5 | 69,224,635 | 58,745,681 | 117,491,362 | 18.04x | 12.29x |
