# Supplementary material for: Target-enriched enzymatic methyl sequencing: Flexible, scalable and inexpensive hybridization capture for quantifying DNA methylation
Source: PLoS One. 2023 Mar 9;18(3):e0282672. doi: 10.1371/journal.pone.0282672 (PMC9997987; doi:10.1371/journal.pone.0282672)
Supplement: S5 Table — (DOCX) [file pone.0282672.s011.docx]

**S5 Table. Summary metrics for reduced-representation bisulfite sequencing (RRBS).**

| **Individual** | **Raw reads** | **Full genome mapping efficiency** | **Full genome unique single-end alignments** | **Mean CpG coverage across genome^*^** | **Mean CpG coverage across putative promoter target regions^**^** |
| --- | --- | --- | --- | --- | --- |
| BB-17532 | 19,253,809 | 52.1 | 9,437,245 | 18.05x | 14.78x |
| BB-17501 | 19,634,960 | 50.1 | 9,504,039 | 18.14x | 14.58x |
| BB-17455 | 14,016,434 | 53.5 | 6,621,698 | 12.80x | 9.51x |
| BB-17411 | 17,805,945 | 55.4 | 7,137,598 | 13.05x | 9.92x |
| BB-17168 | 16,571,855 | 53.8 | 8,649,111 | 16.81x | 13.54x |
| BB-14232 | 19,668,309 | 51.5 | 9,932,118 | 19.40x | 16.03x |
| B-40881 | 29,514,286 | 45.0 | 12,924,898 | 22.87x | 19.18x |

^*^ At 5x coverage or above to avoid low-representation sites in RRBS library.

^**^ Averaging the total count of unmethylated and methylated cytosines for all sites.
