# Supplementary material for: Target-enriched enzymatic methyl sequencing: Flexible, scalable and inexpensive hybridization capture for quantifying DNA methylation
Source: PLoS One. 2023 Mar 9;18(3):e0282672. doi: 10.1371/journal.pone.0282672 (PMC9997987; doi:10.1371/journal.pone.0282672)
Supplement: S6 Table — Libraries for seven of the same individual superb starlings (B-40881, BB-17168, BB-17411, BB-17455, BB-17501, BB-17532, and BB-14232) were sequenced using both methods. The number of shared CpG sites at 5x coverage or above and the mean DNA methylation levels from both methods are shown for each starling sample. Some target regions had very sparse RRBS coverage, resulting in low numbers of shared CpG sites (e.g., ESRI, GNRH1, GNRH2, MC4R, and SERPINA1). See S2 Table for the length of the putative promoter region targeted for each gene. (DOCX) [file pone.0282672.s012.docx]

**S6 Table. Comparison of target-enriched enzymatic methyl sequencing (TEEM-Seq) and reduced-representation bisulfite sequencing (RRBS) in putative promoter regions.** Libraries for seven of the same individual superb starlings (B-40881, BB-17168, BB-17411, BB-17455, BB-17501, BB-17532, and BB-14232) were sequenced using both methods. The number of shared CpG sites at 5x coverage or above and the mean DNA methylation levels from both methods are shown for each starling sample. Some target regions had very sparse RRBS coverage, resulting in low numbers of shared CpG sites (e.g., *ESRI*, *GNRH1*, *GNRH2*, *MC4R*, and *SERPINA1*). See S2 Table for the length of the putative promoter region targeted for each gene.

| **Target region** | **B-40881** | | | **BB-17168** | | | **BB-17411** | | | **BB-17455** | | | **BB-17501** | | | **BB-17532** | | | **BB-14232** | | |
| --- | --- | --- | --- | --- | --- | --- | --- | --- | --- | --- | --- | --- | --- | --- | --- | --- | --- | --- | --- | --- | --- |
|  | **Number shared CpGs** | **TEEM-Seq mean methyl** | **RRBS mean methyl** | **Number shared CpGs** | **TEEM-Seq mean methyl** | **RRBS mean methyl** | **Number shared CpGs** | **TEEM-Seq mean methyl** | **RRBS mean methyl** | **Number shared CpGs** | **TEEM-Seq mean methyl** | **RRBS mean methyl** | **Number shared CpGs** | **TEEM-Seq mean methyl** | **RRBS mean methyl** | **Number shared CpGs** | **TEEM-Seq mean methyl** | **RRBS mean methyl** | **Number shared CpGs** | **TEEM-Seq mean methyl** | **RRBS mean methyl** |
| *AR* | 310 | 1.91 | 2.57 | 207 | 3.76 | 2.87 | 253 | 2.48 | 2.43 | 253 | 2.28 | 3.15 | 221 | 3.15 | 3.17 | 270 | 2.14 | 2.37 | 173 | 4.85 | 3.40 |
| *AVPR1A* | 76 | 1.37 | 1.66 | 76 | 2.19 | 1.39 | 76 | 1.16 | 1.27 | 75 | 1.49 | 1.21 | 76 | 1.60 | 2.28 | 76 | 0.96 | 1.49 | 76 | 3.52 | 1.42 |
| *AVPR1B* | 40 | 49.76 | 50.02 | 43 | 51.27 | 57.37 | 43 | 50.05 | 52.32 | 39 | 50.04 | 50.83 | 28 | 48.29 | 53.71 | 36 | 45.86 | 50.25 | 42 | 47.86 | 45.95 |
| *CRH* | 239 | 1.68 | 1.98 | 190 | 3.44 | 2.28 | 224 | 1.47 | 1.58 | 213 | 2.04 | 1.50 | 234 | 2.14 | 1.95 | 213 | 1.14 | 1.25 | 205 | 3.93 | 2.01 |
| *DNMT1* | 154 | 30.32 | 29.62 | 86 | 13.42 | 13.71 | 115 | 17.51 | 16.04 | 132 | 28.58 | 29.53 | 123 | 25.39 | 24.40 | 120 | 27.82 | 28.16 | 72 | 17.35 | 16.41 |
| *DNMT3B* | 76 | 17.53 | 14.97 | 58 | 10.75 | 6.32 | 58 | 10.18 | 13.40 | 62 | 13.81 | 12.50 | 58 | 14.42 | 13.85 | 70 | 12.73 | 13.47 | 49 | 12.60 | 11.39 |
| *EGR1* | 312 | 0.11 | 0.73 | 304 | 1.55 | 0.68 | 290 | 0.60 | 0.91 | 251 | 0.45 | 0.78 | 283 | 0.33 | 0.75 | 293 | 0.12 | 0.73 | 272 | 1.74 | 0.42 |
| *ESR1* | 0 | - | - | 0 | - | - | 0 | - | - | 0 | - | - | 0 | - | - | 0 | - | - | 0 | - | - |
| *FKBP5* | 6 | 70.61 | 61.41 | 6 | 66.19 | 63.89 | 6 | 66.74 | 66.67 | 6 | 70.27 | 67.05 | 6 | 72.46 | 81.40 | 6 | 71.81 | 67.81 | 6 | 66.18 | 62.88 |
| *GNIH* | 54 | 16.58 | 17.89 | 53 | 15.31 | 10.82 | 51 | 17.19 | 16.88 | 53 | 22.10 | 18.58 | 54 | 17.06 | 14.95 | 50 | 10.37 | 12.60 | 50 | 12.02 | 11.74 |
| *GNRH1* | 0 | - | - | 0 | - | - | 1 | 34.62 | - | 1 | 31.18 | - | 0 | - | - | 0 | - | - | 1 | 25.00 | - |
| *GNRHR2 r1^#^* | 8 | 51.54 | 57.44 | 0 | - | - | 6 | 50.37 | 46.53 | 7 | 54.57 | 44.29 | 8 | 53.76 | 43.45 | 8 | 47.92 | 47.80 | 7 | 48.68 | 59.05 |
| *GNRHR2 r2^#^* | 214 | 41.21 | 41.03 | 185 | 54.39 | 52.21 | 176 | 48.86 | 48.33 | 182 | 48.38 | 47.17 | 200 | 50.17 | 48.59 | 209 | 46.42 | 47.16 | 159 | 52.02 | 50.54 |
| *MC2R* | 161 | 0.67 | 1.25 | 151 | 1.83 | 0.45 | 158 | 0.53 | 0.75 | 161 | 0.79 | 0.95 | 150 | 0.53 | 0.69 | 173 | 0.61 | 0.56 | 146 | 3.78 | 0.39 |
| *MC4R* | 0 | - | - | 0 | - | - | 0 | - | - | 0 | - | - | 0 | - | - | 0 | - | - | 0 | - | - |
| *NR3C1* | 219 | 0.26 | 0.39 | 152 | 1.35 | 0.30 | 195 | 0.24 | 0.29 | 179 | 0.16 | 0.47 | 195 | 0.49 | 0.39 | 220 | 0.17 | 0.36 | 172 | 4.13 | 0.24 |
| *NR3C2* | 486 | 0.32 | 0.33 | 355 | 2.17 | 0.70 | 401 | 0.55 | 0.69 | 412 | 0.58 | 0.70 | 412 | 0.85 | 0.75 | 479 | 0.29 | 0.42 | 363 | 4.44 | 0.47 |
| *OXTR* | 145 | 4.32 | 4.50 | 95 | 9.54 | 3.85 | 120 | 4.38 | 3.82 | 146 | 4.36 | 4.17 | 122 | 6.57 | 5.25 | 125 | 3.55 | 3.19 | 101 | 11.48 | 4.39 |
| *POMC* | 23 | 95.04 | 93.73 | 32 | 76.85 | 82.15 | 39 | 91.95 | 93.59 | 25 | 94.13 | 93.04 | 23 | 96.12 | 93.62 | 33 | 95.08 | 94.75 | 18 | 97.13 | 96.35 |
| *SERPINA1* | 0 | - | - | 0 | - | - | 0 | - | - | 0 | - | - | 0 | - | - | 0 | - | - | 0 | - | - |
| *VT* | 10 | 46.16 | 41.38 | 9 | 55.73 | 46.01 | 10 | 45.33 | 45.74 | 10 | 51.09 | 36.63 | 10 | 48.30 | 48.12 | 9 | 46.66 | 50.84 | 9 | 54.80 | 45.34 |
| *VTG1* | 26 | 8.51 | 6.48 | 21 | 22.95 | 18.80 | 26 | 7.78 | 6.50 | 26 | 7.02 | 2.16 | 22 | 15.48 | 18.03 | 26 | 8.46 | 6.35 | 21 | 8.95 | 11.81 |

^#^ Two separate gene regions (indicated as r1 and r2) on chromosome 10 with similarity to *GNRHR2*.
