# Supplementary material for: Target-enriched enzymatic methyl sequencing: Flexible, scalable and inexpensive hybridization capture for quantifying DNA methylation
Source: PLoS One. 2023 Mar 9;18(3):e0282672. doi: 10.1371/journal.pone.0282672 (PMC9997987; doi:10.1371/journal.pone.0282672)
Supplement: S8 Table — Samples for four of the same individual superb starlings (BB-17411, BB-17455, B-40881, and BB-17532) were made into duplicate (or triplicate for BB-17532) libraries and sequenced. The number of shared CpG sites at 5x coverage or above and the mean DNA methylation levels from each library are shown for each starling sample. Asterisks indicate genes for which more or less than 4000 bp of putative promoter regions were targeted. (DOCX) [file pone.0282672.s014.docx]

**S8 Table. Comparison of samples run as duplicate or triplicate libraries using target-enriched enzymatic methyl sequencing (TEEM-Seq).** Samples for four of the same individual superb starlings (BB-17411, BB-17455, B-40881, and BB-17532) were made into duplicate (or triplicate for BB-17532) libraries and sequenced. The number of shared CpG sites at 5x coverage or above and the mean DNA methylation levels from each library are shown for each starling sample. Asterisks indicate genes for which more or less than 4000 bp of putative promoter regions were targeted.

| **Target region** | **Putative promoter region length (bp)** | **BB-17411** | | | **BB-17455** | | | **B-40881** | | | **BB-17532** | | | | | | | | |
| --- | --- | --- | --- | --- | --- | --- | --- | --- | --- | --- | --- | --- | --- | --- | --- | --- | --- | --- | --- |
|  |  | **Number shared CpGs** | **Library 1 mean methyl** | **Library 2 mean methyl** | **Number shared CpGs** | **Library 1 mean methyl** | **Library 2 mean methyl** | **Number shared CpGs** | **Library 1 mean methyl** | **Library 2 mean methyl** | **Number shared CpGs** | **Library 1 mean methyl** | **Library 2 mean methyl** | **Number shared CpGs** | **Library 1 mean methyl** | **Library 3 mean methyl** | **Number shared CpGs** | **Library 2 mean methyl** | **Library 3 mean methyl** |
| *AR* | 4000 | 430 | 5.10 | 5.18 | 431 | 4.72 | 5.09 | 444 | 4.63 | 4.65 | 377 | 5.29 | 5.28 | 325 | 5.95 | 5.94 | 312 | 6.09 | 6.19 |
| *AVPR1A* | 4000 | 152 | 16.39 | 16.26 | 151 | 16.06 | 15.84 | 154 | 16.58 | 15.93 | 152 | 15.18 | 14.27 | 150 | 15.16 | 15.21 | 150 | 14.09 | 15.21 |
| *AVPR1B* | 4000 | 125 | 45.47 | 44.64 | 123 | 45.04 | 46.47 | 124 | 43.77 | 44.55 | 123 | 42.03 | 41.49 | 117 | 42.80 | 40.31 | 117 | 41.93 | 40.31 |
| *CRH* | 4000 | 364 | 4.37 | 4.27 | 370 | 5.18 | 5.71 | 372 | 4.57 | 4.86 | 357 | 4.24 | 4.18 | 334 | 4.35 | 5.05 | 333 | 4.23 | 5.07 |
| *DNMT1* | 4000 | 286 | 50.07 | 48.21 | 288 | 47.54 | 47.37 | 301 | 46.90 | 46.72 | 247 | 55.90 | 54.81 | 211 | 61.23 | 60.66 | 211 | 60.18 | 60.66 |
| *DNMT3A* | 4000 | 439 | 78.10 | 77.33 | 473 | 77.95 | 76.62 | 513 | 79.37 | 79.64 | 418 | 79.40 | 79.90 | 366 | 79.26 | 78.25 | 353 | 79.68 | 78.06 |
| *DNMT3B* | 4000 | 183 | 25.19 | 25.21 | 183 | 25.60 | 25.24 | 183 | 26.60 | 26.26 | 183 | 23.03 | 23.25 | 183 | 23.03 | 23.61 | 183 | 23.25 | 23.61 |
| *EGR1* | 4000 | 456 | 2.63 | 2.52 | 458 | 2.84 | 2.55 | 472 | 2.41 | 2.62 | 459 | 2.31 | 2.59 | 415 | 2.53 | 3.72 | 415 | 2.83 | 3.72 |
| *ESR1* | 4000 | 48 | 58.81 | 55.23 | 47 | 59.74 | 62.25 | 55 | 57.44 | 56.77 | 50 | 59.39 | 61.09 | 44 | 59.18 | 61.74 | 44 | 61.23 | 61.74 |
| *FKBP5* | 4000 | 103 | 40.30 | 38.22 | 103 | 43.50 | 43.20 | 104 | 41.62 | 41.68 | 103 | 39.88 | 37.65 | 102 | 40.27 | 40.59 | 102 | 38.02 | 40.59 |
| *GNIH* | 4000 | 129 | 28.68 | 26.61 | 128 | 31.30 | 34.05 | 131 | 28.15 | 28.34 | 129 | 23.85 | 24.07 | 127 | 23.91 | 23.14 | 127 | 24.01 | 23.14 |
| *GNRH1* | 5726* | 22 | 45.52 | 48.92 | 22 | 41.15 | 41.98 | 24 | 42.94 | 42.50 | 23 | 40.31 | 38.39 | 21 | 41.18 | 43.82 | 21 | 39.23 | 43.82 |
| *GNRHR2 (r1)*^#^ | 4000 | 85 | 47.92 | 48.84 | 83 | 47.68 | 48.99 | 89 | 44.75 | 44.73 | 84 | 45.35 | 44.93 | 82 | 46.45 | 45.93 | 82 | 46.02 | 45.93 |
| *GNRHR2 (r2)*^#^ | 4000 | 388 | 51.86 | 49.94 | 371 | 50.45 | 50.86 | 389 | 48.30 | 48.91 | 370 | 49.85 | 51.30 | 327 | 54.42 | 55.53 | 321 | 55.60 | 55.63 |
| *MC2R* | 3856 | 279 | 4.62 | 4.97 | 279 | 4.81 | 5.01 | 293 | 5.09 | 5.04 | 279 | 4.59 | 5.71 | 279 | 4.59 | 4.54 | 272 | 5.86 | 4.65 |
| *MC4R* | 3948* | 32 | 42.24 | 38.52 | 31 | 41.43 | 40.61 | 32 | 43.63 | 41.79 | 32 | 41.10 | 39.70 | 32 | 41.10 | 37.45 | 32 | 39.70 | 37.45 |
| *NR3C1* | 4000 | 333 | 0.43 | 0.30 | 314 | 0.31 | 0.22 | 392 | 0.22 | 0.25 | 316 | 0.20 | 0.25 | 210 | 0.27 | 0.16 | 206 | 0.17 | 0.06 |
| *NR3C2* | 4000 | 756 | 2.21 | 2.21 | 752 | 2.35 | 2.05 | 824 | 1.96 | 1.91 | 719 | 1.90 | 2.45 | 589 | 2.27 | 2.77 | 560 | 2.87 | 2.70 |
| *OXTR* | 4000 | 228 | 15.91 | 17.68 | 204 | 18.45 | 19.51 | 244 | 15.80 | 16.57 | 205 | 16.34 | 17.21 | 180 | 17.96 | 20.64 | 197 | 17.20 | 18.96 |
| *POMC* | 258** | 32 | 92.09 | 92.61 | 23 | 93.37 | 94.52 | 48 | 93.50 | 93.16 | 42 | 94.47 | 91.35 | 34 | 95.19 | 93.79 | 31 | 90.65 | 93.19 |
| *SERPINA1* | 4000 | 15 | 44.88 | 45.92 | 13 | 53.40 | 47.63 | 16 | 47.16 | 46.39 | 15 | 46.63 | 42.52 | 14 | 46.69 | 41.69 | 14 | 42.70 | 41.69 |
| *VT* | 4000 | 133 | 37.30 | 35.46 | 133 | 38.46 | 40.57 | 137 | 37.73 | 37.80 | 130 | 34.25 | 34.27 | 128 | 34.43 | 33.80 | 126 | 34.40 | 33.92 |
| *VTG1* | 697** | 56 | 9.77 | 7.91 | 55 | 8.11 | 8.46 | 70 | 7.23 | 7.64 | 55 | 8.66 | 10.08 | 40 | 10.24 | 9.74 | 40 | 13.51 | 9.74 |

^*^ Since there is no existing annotation in the superb starling reference genome for this gene, we used the zebra finch sequence alignment to target 2 kb upstream in the putative promoter region and 2 kb in the gene body.

^**^ Limited sequence upstream of gene.

^#^ Two separate gene regions (indicated as r1 and r2) on chromosome 10 with similarity to *GNRHR2*.
