# Supplementary material for: Target-enriched enzymatic methyl sequencing: Flexible, scalable and inexpensive hybridization capture for quantifying DNA methylation
Source: PLoS One. 2023 Mar 9;18(3):e0282672. doi: 10.1371/journal.pone.0282672 (PMC9997987; doi:10.1371/journal.pone.0282672)
Supplement: S9 Table — Libraries for three of the same individual superb starlings (BB-17168, BB-17501, and BB-14232) were sequenced using both methods. The number of shared CpG sites at 5x coverage or above and the mean DNA methylation levels from both methods are shown for each starling sample. (DOCX) [file pone.0282672.s015.docx]

**S9 Table. Comparison of whole-genome enzymatic methyl sequencing (WGEM-Seq) and target-enriched enzymatic methyl sequencing (TEEM-Seq) in exonic regions.** Libraries for three of the same individual superb starlings (BB-17168, BB-17501, and BB-14232) were sequenced using both methods. The number of shared CpG sites at 5x coverage or above and the mean DNA methylation levels from both methods are shown for each starling sample.

| **Target region** | **BB-17168** | | | **BB-17501** | | | **BB-14232** | | |
| --- | --- | --- | --- | --- | --- | --- | --- | --- | --- |
|  | **Number shared CpGs** | **WGEM-Seq mean methyl** | **TEEM-Seq mean methyl** | **Number shared CpGs** | **WGEM-Seq mean methyl** | **TEEM-Seq mean methyl** | **Number shared CpGs** | **WGEM-Seq mean methyl** | **TEEM-Seq mean methyl** |
| *AR* | 151 | 32.45 | 31.65 | 163 | 36.96 | 37.46 | 103 | 40.21 | 40.34 |
| *AVPR1A* | 229 | 8.42 | 7.06 | 172 | 9.03 | 8.21 | 115 | 9.19 | 10.20 |
| *AVPR1B* | 111 | 66.61 | 68.59 | 116 | 64.81 | 64.81 | 72 | 80.31 | 76.70 |
| *CRH* | 41 | 2.32 | 0.69 | 40 | 0.00 | 0.17 | 15 | 6.41 | 3.31 |
| *EGR1* | 155 | 21.54 | 20.53 | 158 | 25.03 | 25.06 | 120 | 25.57 | 25.76 |
| *FKBP5* | 66 | 64.57 | 63.02 | 60 | 67.84 | 64.85 | 31 | 68.63 | 68.82 |
| *GNRHR2 r1*^#^ | 53 | 70.84 | 70.77 | 62 | 72.68 | 73.50 | 39 | 68.13 | 68.07 |
| *GNRHR2 r2*^#^ | 120 | 76.15 | 76.49 | 130 | 78.31 | 78.39 | 89 | 78.93 | 82.03 |
| *NR3C1* | 260 | 57.74 | 58.36 | 241 | 57.52 | 60.04 | 119 | 47.73 | 51.44 |
| *NR3C2* | 145 | 85.30 | 82.91 | 141 | 85.63 | 85.53 | 84 | 83.30 | 82.98 |
| *OXTR* | 65 | 55.26 | 56.32 | 58 | 69.73 | 67.39 | 42 | 55.55 | 60.35 |
| *POMC* | 22 | 88.21 | 88.18 | 26 | 86.28 | 87.31 | 21 | 92.18 | 90.93 |

^#^ Two separate gene regions (indicated as r1 and r2) on chromosome 10 with similarity to *GNRHR2*.
