# Supplementary material for: Target-enriched enzymatic methyl sequencing: Flexible, scalable and inexpensive hybridization capture for quantifying DNA methylation
Source: PLoS One. 2023 Mar 9;18(3):e0282672. doi: 10.1371/journal.pone.0282672 (PMC9997987; doi:10.1371/journal.pone.0282672)
Supplement: S10 Table — WGEM-Seq and RRBS data were compared in 20 random 2.5 kb “promoter” regions that were not included in the bait set because we expected that some RRBS regions corresponding to target ranges might have sparse coverage in comparison to the high coverage bait alignments. The position of each random 2.5 kb “promoter” region in the superb starling genome (Lasu_v2), the chromosome number, and “promoter” orientation (‘rev’ indicates ‘reversed’). Libraries for three of the same individual superb starlings (BB-17168, BB-17501, and BB-14232) were sequenced using both methods. The sequence start and ending locations, the number of shared CpG sites at 5x coverage or above, and the mean DNA methylation levels from both methods are shown for each starling sample. (DOCX) [file pone.0282672.s016.docx]

**S10 Table. Comparison of whole-genome enzymatic methyl sequencing (WGEM-Seq) and reduced-representation bisulfite sequencing (RRBS) in random 2.5 kb “promoter” regions.** WGEM-Seq and RRBS data were compared in 20 random 2.5 kb “promoter” regions that were not included in the bait set because we expected that some RRBS regions corresponding to target ranges might have sparse coverage in comparison to the high coverage bait alignments. The position of each random 2.5 kb “promoter” region in the superb starling genome (Lasu_v2), the chromosome number, and “promoter” orientation (‘rev’ indicates ‘reversed’). Libraries for three of the same individual superb starlings (BB-17168, BB-17501, and BB-14232) were sequenced using both methods. The sequence start and ending locations, the number of shared CpG sites at 5x coverage or above, and the mean DNA methylation levels from both methods are shown for each starling sample.

| **Genome position** | **BB-17168** | | | | | | **BB-17501** | | | | | | **BB-14232** | | | | | |
| --- | --- | --- | --- | --- | --- | --- | --- | --- | --- | --- | --- | --- | --- | --- | --- | --- | --- | --- |
| ***Chromosome*** | **Start sequence location (1-based)** | **End sequence location** | **Length (bp)** | **Number shared CpGs** | **WGEM-Seq mean**  **methyl** | **RRBS mean**  **methyl** | **Start sequence location (1-based)** | **End sequence location** | **Length (bp)** | **Number shared CpGs** | **WGEM-Seq mean**  **methyl** | **RRBS mean**  **methyl** | **Start sequence location (1-based)** | **End sequence location** | **Length (bp)** | **Number shared CpGs** | **WGEM-Seq mean**  **methyl** | **RRBS mean**  **methyl** |
| CM040302.1  *chr 1* | 33752942 | 33753769 | 828 | 62 | 0.55 | 0.42 | 33752942 | 33753751 | 810 | 70 | 0.00 | 0.27 | 33753270 | 33753768 | 499 | 59 | 2.67 | 0.53 |
| CM040302.1  *chr 1 (rev)* | 62786630 | 62787143 | 514 | 28 | 1.00 | 0.25 | 62786427 | 62787143 | 717 | 55 | 0.61 | 0.16 | 62786638 | 62787130 | 493 | 29 | 4.52 | 0.13 |
| CM040303.1  *chr 1A* | 27626923 | 27627048 | 126 | 14 | 0.00 | 0.79 | 27626860 | 27627068 | 209 | 21 | 0.00 | 0.40 | 27626922 | 27627048 | 127 | 0 | N/A | N/A |
| CM040303.1  *chr 1A (rev) r1 ^#^* | 10576313 | 10577590 | 1278 | 59 | 2.22 | 3.19 | 10576294 | 10577591 | 1298 | 52 | 0.00 | 1.84 | 10576289 | 10577633 | 1345 | 88 | 1.84 | 0.69 |
| CM040303.1  *chr 1A (rev) r2 ^#^* | 39265876 | 39267417 | 1542 | 134 | 0.25 | 0.28 | 39265876 | 39267417 | 1542 | 219 | 0.00 | 0.40 | 39265911 | 39267426 | 1516 | 177 | 4.38 | 0.24 |
| CM040305.1  *chr 3 r1^#^* | 12188281 | 12189069 | 789 | 37 | 2.05 | 1.19 | 12188282 | 12189069 | 788 | 26 | 1.28 | 2.21 | 12188282 | 12189082 | 801 | 20 | 10.33 | 3.36 |
| CM040305.1  *chr 3 r2^#^* | 43713632 | 43713963 | 332 | 37 | 4.11 | 0.12 | 43713632 | 43713963 | 332 | 21 | 0.00 | 0.25 | 43713663 | 43713963 | 301 | 7 | 0.00 | 1.02 |
| CM040306.1  *chr 4 (rev)* | 18836665 | 18837815 | 1151 | 71 | 5.04 | 0.83 | 18836672 | 18837050 | 379 | 13 | 0.00 | 0.00 | 18836881 | 18837814 | 934 | 62 | 7.37 | 1.44 |
| CM040307.1  *chr 4A (rev)* | 9285901 | 9287354 | 1454 | 60 | 3.89 | 4.33 | 9285901 | 9287355 | 1455 | 34 | 8.50 | 6.42 | 9287200 | 9287368 | 169 | 29 | 2.53 | 0.39 |
| CM040308.1  *chr 5A* | 30585721 | 30585808 | 88 | 10 | 2.43 | 13.00 | 30585691 | 30585808 | 118 | 19 | 24.78 | 23.82 | 30585707 | 30585809 | 103 | 21 | 10.98 | 2.73 |
| CM040310.1  *chr 6A* | 12600601 | 12601812 | 1212 | 10 | 28.44 | 25.18 | 12600601 | 12601812 | 1212 | 9 | 35.41 | 31.13 | 12601734 | 12601811 | 78 | 4 | 2.08 | 1.92 |
| CM040312.1  *chr 7 (rev)* | 26600657 | 26601613 | 957 | 54 | 2.31 | 1.71 | 26600729 | 26601612 | 884 | 79 | 0.75 | 0.86 | 26600998 | 26601403 | 406 | 60 | 6.17 | 0.14 |
| CM040317.1  *chr 12* | 518243 | 518850 | 608 | 32 | 2.41 | 1.42 | 518244 | 518926 | 683 | 13 | 0.00 | 0.96 | 518251 | 518809 | 559 | 20 | 11.24 | 0.66 |
| CM040322.1  *chr 18 (rev)* | 11611593 | 11612416 | 824 | 65 | 11.80 | 11.72 | 11611593 | 11612417 | 825 | 47 | 12.75 | 10.72 | 11611593 | 11612429 | 837 | 69 | 6.34 | 4.75 |
| CM040325.1  *chr 21 (rev)* | 641318 | 641744 | 427 | 13 | 4.18 | 0.59 | 641317 | 641505 | 189 | 24 | 0.83 | 0.21 | 641314 | 641506 | 193 | 32 | 7.50 | 0.63 |
| CM040327.1  *chr 23* | 7414348 | 7415383 | 1036 | 24 | 0.83 | 0.00 | 7414415 | 7415713 | 1299 | 14 | 1.43 | 1.43 | 7414918 | 7415028 | 111 | 25 | 1.60 | 0.30 |
| CM040329.1  *chr 25* | 3307103 | 3308528 | 1426 | 52 | 9.76 | 7.90 | 3307138 | 3308528 | 1391 | 57 | 14.58 | 13.62 | 3307852 | 3308095 | 244 | 28 | 3.37 | 0.11 |
| CM040330.1  *chr 26 (rev)* | 1656448 | 1657477 | 1030 | 41 | 13.25 | 11.71 | 1656887 | 1657532 | 646 | 19 | 39.37 | 30.22 | 1656256 | 1657478 | 1223 | 49 | 11.89 | 5.13 |
| CM040333.1  *chr 29* | 2390588 | 2392092 | 1505 | 35 | 0.00 | 0.00 | 2390589 | 2392092 | 1504 | 39 | 0.51 | 0.52 | 2390750 | 2390868 | 119 | 5 | 0.00 | 0.00 |
| CM040335.1  *chr Z* | 20569346 | 20569391 | 46 | 9 | 0.00 | 1.85 | 20568984 | 20569391 | 408 | 34 | 0.42 | 0.35 | 20569345 | 20569370 | 26 | 7 | 4.76 | 0.00 |

^#^ Two separate gene regions (indicated as r1 and r2) on chromosome 1A (rev) and chromosome 3.
