# Supplementary material for: Target-enriched enzymatic methyl sequencing: Flexible, scalable and inexpensive hybridization capture for quantifying DNA methylation
Source: PLoS One. 2023 Mar 9;18(3):e0282672. doi: 10.1371/journal.pone.0282672 (PMC9997987; doi:10.1371/journal.pone.0282672)
Supplement: S11 Table — Libraries for three of the same individual superb starlings (BB-17168, BB-17501, and BB-14232) were sequenced using both methods. The number of shared CpG sites at 5x coverage or above and the mean DNA methylation levels from both methods are shown for each starling sample. WGEM-Seq data is deduplicated by default. S7 Table shows the same comparison for TEEM-Seq data with deduplication. Given the qualitatively similar results using deduplication and non-deduplication of TEEM-Seq data, similar to RRBS analysis, deduplication may not be necessary for TEEM-Seq analysis. Asterisks indicate genes for which more or less than 4000 bp of putative promoter regions were targeted. (DOCX) [file pone.0282672.s017.docx]

**S11 Table. Comparison of whole-genome enzymatic methyl sequencing (WGEM-Seq) and target-enriched enzymatic methyl sequencing (TEEM-Seq) in putative promoter regions for TEEM-Seq data without deduplication.** Libraries for three of the same individual superb starlings (BB-17168, BB-17501, and BB-14232) were sequenced using both methods. The number of shared CpG sites at 5x coverage or above and the mean DNA methylation levels from both methods are shown for each starling sample. WGEM-Seq data is deduplicated by default. S7 Table shows the same comparison for TEEM-Seq data with deduplication. Given the qualitatively similar results using deduplication and non-deduplication of TEEM-Seq data, similar to RRBS analysis, deduplication may not be necessary for TEEM-Seq analysis. Asterisks indicate genes for which more or less than 4000 bp of putative promoter regions were targeted.

| **Target region** | **Putative promoter region length (bp)** | **BB-17168** | | | **BB-17501** | | | **BB-14232** | | |
| --- | --- | --- | --- | --- | --- | --- | --- | --- | --- | --- |
|  |  | **Number shared CpGs** | **WGEM-Seq mean methyl** | **TEEM-Seq mean methyl** | **Number shared CpGs** | **WGEM-Seq mean methyl** | **TEEM-Seq mean**  **methyl** | **Number shared CpGs** | **WGEM-Seq mean methyl** | **TEEM-Seq mean methyl** |
| *AR* | 4000 | 212 | 9.45 | 8.76 | 226 | 10.27 | 9.30 | 117 | 13.87 | 11.75 |
| *AVPR1A* | 4000 | 60 | 31.17 | 32.96 | 104 | 23.29 | 22.01 | 48 | 18.49 | 18.24 |
| *AVPR1B* | 4000 | 83 | 48.45 | 48.08 | 67 | 40.56 | 41.63 | 53 | 41.89 | 41.99 |
| *CRH* | 4000 | 251 | 7.08 | 6.10 | 239 | 4.58 | 4.68 | 187 | 10.21 | 8.56 |
| *DNMT1* | 4000 | 105 | 40.79 | 42.10 | 131 | 51.64 | 50.57 | 35 | 81.18 | 83.86 |
| *DNMT3A* | 4000 | 288 | 80.64 | 80.78 | 329 | 81.09 | 80.08 | 177 | 83.52 | 82.58 |
| *DNMT3B* | 4000 | 149 | 26.39 | 25.96 | 125 | 33.51 | 34.84 | 98 | 27.97 | 24.37 |
| *EGR1* | 4000 | 328 | 5.47 | 4.78 | 290 | 3.93 | 3.84 | 155 | 7.11 | 6.14 |
| *ESR1* | 4000 | 48 | 55.68 | 58.61 | 44 | 63.91 | 62.57 | 23 | 59.84 | 55.63 |
| *FKBP5* | 4000 | 77 | 40.30 | 39.85 | 78 | 47.61 | 45.59 | 46 | 41.99 | 39.25 |
| *GNIH* | 4000 | 108 | 29.63 | 27.98 | 109 | 28.57 | 28.95 | 71 | 27.31 | 31.62 |
| *GNRH1* | 5726* | 16 | 40.23 | 42.88 | 18 | 45.00 | 50.10 | 4 | 39.40 | 37.41 |
| *GNRHR2 r1*^#^ | 4000 | 70 | 46.24 | 47.89 | 66 | 53.62 | 49.85 | 50 | 46.46 | 43.08 |
| *GNRHR2 r2*^#^ | 4000 | 189 | 62.80 | 64.01 | 196 | 60.59 | 60.38 | 112 | 58.82 | 57.18 |
| *MC2R* | 3856 | 194 | 10.18 | 7.94 | 143 | 8.57 | 9.37 | 39 | 17.58 | 19.16 |
| *MC4R* | 3948* | 26 | 38.59 | 43.01 | 28 | 39.68 | 39.57 | 21 | 36.13 | 43.00 |
| *NR3C1* | 4000 | 108 | 0.55 | 0.42 | 172 | 0.56 | 0.34 | 141 | 2.11 | 1.39 |
| *NR3C2* | 4000 | 194 | 8.18 | 8.22 | 176 | 6.79 | 8.79 | 33 | 8.24 | 6.44 |
| *OXTR* | 4000 | 103 | 21.72 | 22.96 | 116 | 22.02 | 22.41 | 69 | 29.88 | 28.38 |
| *POMC* | 258** | 20 | 81.25 | 76.16 | 9 | 94.71 | 96.31 | 4 | 100.00 | 98.81 |
| *SERPINA1* | 4000 | 13 | 55.74 | 56.68 | 12 | 44.55 | 43.07 | 12 | 46.83 | 45.02 |
| *VT* | 4000 | 54 | 48.51 | 46.11 | 79 | 44.55 | 40.16 | 78 | 29.51 | 33.89 |
| *VTG1* | 697** | 37 | 25.20 | 19.93 | 13 | 20.00 | 21.50 | 15 | 24.25 | 13.98 |

^*^ Since there is no existing annotation in the superb starling reference genome for this gene, we used the zebra finch sequence alignment to target 2 kb upstream in the putative promoter region and 2 kb in the gene body.

^**^ Limited sequence upstream of gene.

^#^ Two separate gene regions (indicated as r1 and r2) on chromosome 10 with similarity to *GNRHR2*.
