# Supplementary material for: Target-enriched enzymatic methyl sequencing: Flexible, scalable and inexpensive hybridization capture for quantifying DNA methylation
Source: PLoS One. 2023 Mar 9;18(3):e0282672. doi: 10.1371/journal.pone.0282672 (PMC9997987; doi:10.1371/journal.pone.0282672)
Supplement: S12 Table — (DOCX) [file pone.0282672.s018.docx]

**S12 Table. Summary of differences in approach and cost, as well as the pros and cons, of whole-genome enzymatic methyl sequencing (WGEM-Seq), reduced-representation bisulfite sequencing (RRBS), and target-enriched enzymatic methyl sequencing (TEEM-Seq).**

|  | **WGEM-Seq** | **RRBS** | **TEEM-Seq** |
| --- | --- | --- | --- |
| **Input DNA*** | 80-100 ng | 100 ng | 80-100 ng |
| **Percent CpG sites covered** | 90-95% | 10-15% | user defined |
| **Number samples per reaction** | 1 sample | 1 sample | up to 96 samples |
| **Average sequencing depth per sample** | 27.5 Gb | 1.75 Gb | 2.5 Gb |
| **Preparation time (for 96 samples)** | 4 days | 3 days | 5 days |
| **Per sample costs (as of 2022)** |  |  |  |
| **Reagents^**^** | $40.00 | $135.00 | $10.00 |
| **Sequencing^#^** | $275.00 | $17.50 | $25.00 |
| **TOTAL** | $315.00 | $152.50 | $35.00 |
| **Pros** | Targets nearly all CpG sites in genome | Targets ~10-15% of CpG sites in genome | Near-complete coverage for targeted regions |
|  |  | Good coverage in putative promoter regions | No bias toward CpG rich regions |
| **Cons** | Expensive per sample cost | CpG sites can be missed outside of CpG islands | Longer prep time |
|  |  | Bisulfite treatment fragments DNA and results in GC bias | High read duplicate rate |

^*^ Based on NEB and NuGEN kits. EM-Seq can theoretically be used down to 10 ng.

^**^ Includes kits (for WGEM-Seq, RRBS, and TEEM-Seq), other reagents (extra beads and probes for TEEM-Seq), sample quality control (for WGEM-Seq, RRBS, and TEEM-Seq), and DNA shearing (for WGEM-Seq and TEEM-Seq). RRBS prices could be reduced using other types of commercial kits or protocols.

^#^ Assuming $10.00 per Gb for 150 PE.
